# New therapies in celiac disease

**DOI:** 10.1097/MOG.0000000000001080

**Published:** 2025-01-27

**Authors:** Antonella Santonicola, Carlo Soldaini, Carolina Ciacci

**Affiliations:** Gastrointestinal Unit, Department of Medicine, Surgery and Dentistry “Scuola Medica Salernitana”, University of Salerno, Salerno, Italy

**Keywords:** celiac disease, drug development, gluten-free diet, new therapies, pathophysiology

## Abstract

**Purpose of review:**

Celiac disease (CeD) is a chronic autoimmune disorder of the small intestine triggered by gluten ingestion in genetically predisposed individuals. The cornerstone of CeD management remains a strict adherence to a lifelong gluten-free diet (GFD), although such a dietary restriction can lead to an altered quality of life and may not be easy to follow for many patients. These challenges highlighted the need for alternative therapies. This review aims to explore the latest advancements in these therapeutic avenues, emphasizing mechanisms of action, clinical efficacy, and safety profiles of drugs currently in advanced stages of clinical testing.

**Recent findings:**

Recent advances in the understanding of CeD pathophysiology have catalyzed the development of new therapeutic approaches, which include strategies to modify gluten processing in the gut, block gluten-triggered immune responses, or restore immune tolerance to gluten.

**Summary:**

While these therapies are not poised to take the place of GFD, they represent promising treatment alternatives that could enhance the quality of life and minimize long-term consequences in CeD patients. Further research, as well as phase III clinical trials of those already conducted, are needed to establish the feasibility of integrating these novel drugs in the clinical management of CeD.

## Graphical abstract

**Figure:**
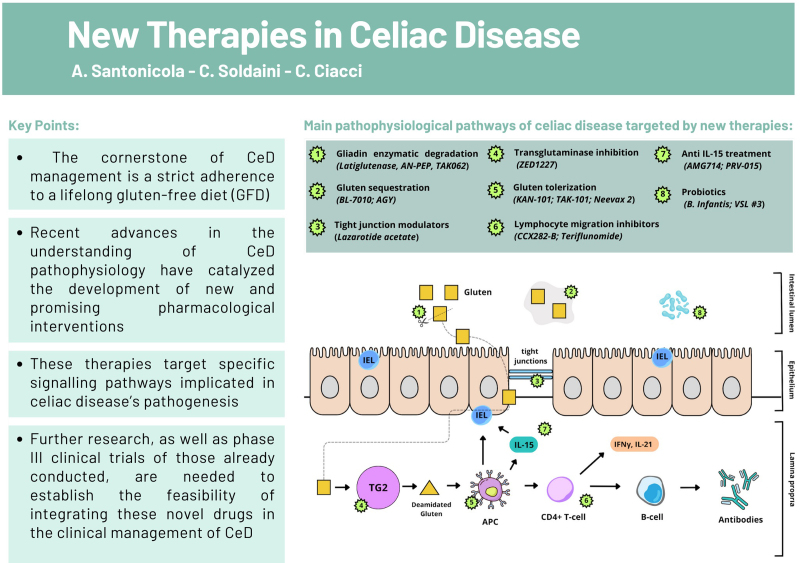

## INTRODUCTION

Celiac disease is a chronic autoimmune disorder of the small intestine triggered by gluten ingestion in genetically predisposed individuals [1]. It affects approximately 1.4% of the global population, with its prevalence rising worldwide [2]. The disease results from a complex interaction between genetic susceptibility and environmental factors, particularly gluten, leading to intestinal inflammation and villous atrophy. CeD could manifest with gastrointestinal symptoms such as abdominal pain, diarrhea, and weight loss. Increasing attention has also been given to its extra-intestinal symptoms, including anemia, osteoporosis, and liver damage [3^▪^].

Since CeD was first described, the only effective treatment for CeD has been a strict, lifelong gluten-free diet (GFD). However, adhering to this diet is often challenging and may not completely prevent inflammatory responses or associated complications. Moreover, dietary restrictions might impact daily life and negatively affect social interactions and quality of life [4]. In the last years, the treatment landscape for CeD has been expanding. This evolution is driven by a deeper understanding of the disease's pathophysiology and the introduction of promising pharmacological interventions.

This review highlights the emerging therapeutic approaches for CeD, focusing on drugs currently in advanced stages of clinical testing. These therapies target specific signaling pathways implicated in the disease's pathogenesis. Additionally, we explore how these treatments could change CeD management by providing alternatives to the dietary approach in order to improve patient outcomes and enhance their quality of life. 

**Box 1:**
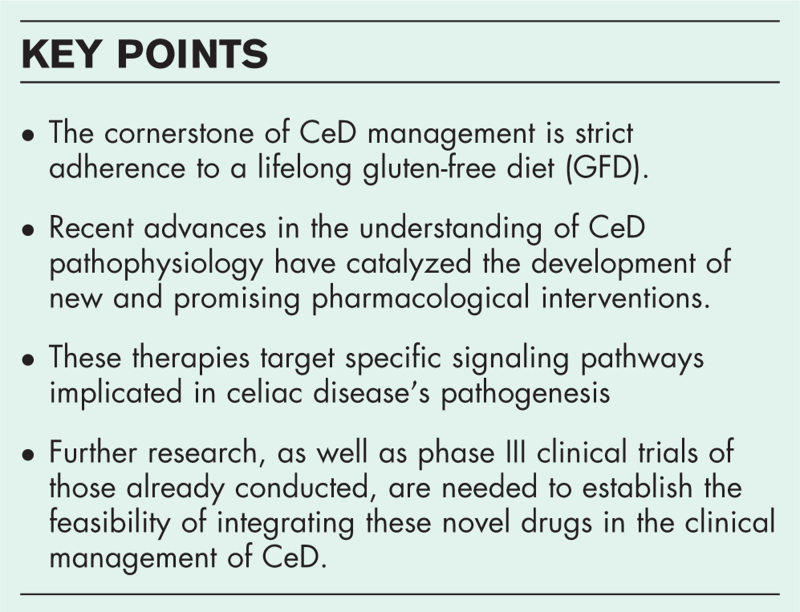
no caption available

## PATHOPHYSIOLOGY

Genetically, CeD is strongly associated with the HLA-DQ2 or HLA-DQ8 haplotypes, positive in the majority of patients and encoded by genes located on chromosome 6 [5]. These molecules are expressed on antigen-presenting cells (APCs) and play a central role in celiac disease pathogenesis by presenting HLA-gluten-polypeptide complexes to CD4^+^ T cells.

Gluten is the commonly used term for the complex of water insoluble proteins from wheat, rye and barley that are harmful to patients with celiac disease.

Wheat gluten, whose proteins represent the CeD antigens, is composed of two main protein types: gliadins and glutenins. Gliadin, due to its high proline and glutamine content, is resistant to complete digestion by gastrointestinal enzymes [6]. This leads to the persistence of large immunogenic peptides, such as the 33-mer peptide derived directly from gliadin, which resist proteolysis and remain in the intestinal lumen [7]. These peptides can traverse the intestinal epithelial barrier, a process facilitated by increased epithelial permeability in CeD, potentially caused by the upregulation of zonulin or the activity of intraepithelial lymphocytes (IELs) [8]. Once in the lamina propria, gluten peptides are deamidated by tissue transglutaminase 2 (TG2), a process that converts specific glutamine residues to glutamic acid, increasing the binding affinity of these peptides to HLA-DQ2 or DQ8 molecules. This leads to the activation of gluten-specific CD4^+^ T cells, which in turn stimulate CD8^+^ T cells, leading to secrete pro-inflammatory cytokines such as interferon-gamma (IFN-γ) and interleukin-21 (IL-21) [9]. These cytokines drive inflammation, damage the intestinal epithelium, and contribute to crypt hyperplasia and villous atrophy, hallmarks of CeD pathology. Innate immune system also plays a crucial role in CeD pathogenesis. IL-15 is overexpressed in the intestinal epithelium and lamina propria of CeD patients, promoting the activation of IELs [10]. These IELs express natural killer (NK) cell receptors, such as NKG2D, and target intestinal epithelial cells (IECs) that express its ligand, MICA, leading to their destruction [10]. This cytotoxic activity exacerbates epithelial barrier dysfunction and enhances gluten peptide translocation.

**FIGURE 1:**
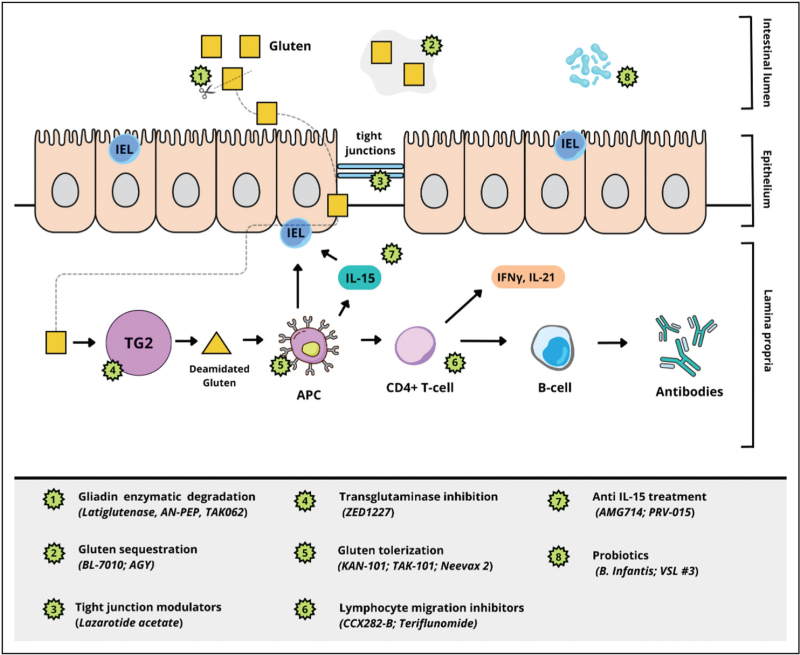
Main pathophysiological mechanisms of celiac disease and new therapeutic approaches that have been proposed (original figure); APC, antigen-presenting cells; IELs, intraepithelial lymphocytes; IFN-γ, Interferon-γ; IL, interleukin; TG2, transglutaminase-2.

Furthermore, gluten-specific T cells stimulate B cells to produce autoantibodies against TG2 and deamidated gluten peptides, which serve as diagnostic markers of CeD [11]. The chronic inflammatory setting, driven by both innate and adaptive immune responses, contributes to tissue destruction, characterized by an increase in IELs, crypt hyperplasia, and villous shortening. Figure 1 summarizes the cascade of events that characterize CeD pathophysiology. These changes impair nutrient absorption and underlie the clinical manifestations of CeD, ranging from gastrointestinal symptoms to systemic complications such as anemia and osteoporosis.

Emerging evidence suggests that environmental factors, including gastrointestinal infections, may also contribute to CeD pathogenesis by triggering immune pathways that increase intestinal permeability or alter immune regulation [12].

A deeper understanding of this complex interplay of genetic predisposition, gluten exposure, immune dysregulation, and environmental triggers highlights the multifaceted nature of CeD pathophysiology and the possibility of new and advanced therapies (Fig. 1).

## EMERGING THERAPEUTIC APPROACHES

### Gliadin enzymatic degradation

Several studies focused on gluten sequestration and enzymatic degradation to reduce the impact of gluten exposure in individuals with CeD.

*Aspergillus niger* derived prolyl endoprotease (AN-PEP) is an oral enzyme capable of breaking down gluten's antigenic proteins, thereby reducing their immunogenicity and preventing intestinal injury [13]. This enzyme functions within a pH range of 2–8, making it resistant to pepsin and suitable for oral administration without requiring special handling. AN-PEP's activity might be influenced by dietary factors: for example, acidic environments enhance its effectiveness, whereas food proteins decrease it [14]. In trial NCT00810654, involving 16 adults, AN-PEP was well tolerated, with no serious adverse effects [15]. The study reported stable histological and immunological findings among participants consuming gluten and AN-PEP, suggesting its safety. However, a recent clinical study [16^▪^] showed that AN-PEP treatment did not significantly lower the total concentration of gluten immunogenic peptides (GIPs) in stool compared to placebo. However, a reduction in severe symptoms was reported among treated patients, highlighting its potential as a supplement to a GFD. However, the limited evidence on AN-PEP precludes drowning conclusions about its efficacy.

Latiglutenase (ALV003) is an experimental oral drug comprising two recombinant proteases: ALV001 and ALV002. ALV001 is a cysteine endoprotease B-isoform, while ALV002 is a prolyl endopeptidase. These enzymes work synergistically to break down immunogenic gluten peptides. ALV003 has demonstrated safety in clinical studies, with no significant adverse reactions reported, even at doses as high as 1800 mg. Patients also showed improvements in symptoms and quality of life. Research indicates that Latiglutenase protects the intestinal mucosa, reduces GIP levels in urine, and alleviates symptoms. Its mechanism involves the sequential action of its enzymes: ALV001 targets specific sequences in the 31–43 and 33-mer peptides, breaking them into smaller fragments, while ALV002 cleaves proline-glutamine bonds, rendering the peptides nonimmunogenic [17]. In the NCT01917630 Phase 2b trial, different doses of ALV003 were evaluated over 12 weeks. While no significant changes were observed in the primary endpoint (villous height-to-crypt depth ratio) or serologic markers, a post hoc analysis of autoantibody-positive patients revealed a dose-dependent reduction in symptom severity. Other trials, such as NCT01255696 and NCT00959114, demonstrated ALV003's ability to mitigate gluten-induced intestinal damage and reduce CD3+ IELs compared to placebo during gluten challenges [18]. Moreover, there are several ongoing Phase 2 trials [19]:

(1) NCT03585478 assesses a 1200 mg dose in patients consuming 2 g of gluten daily for 6 weeks. The treatment reduced villous atrophy and symptom severity compared to placebo.
(2) NCT04243551 is a crossover trial in symptomatic patients adhering to a GFD, which has completed but not yet reported its results.
(3) NCT04839575: evaluates latiglutenase in patients with type 1 diabetes and CeD, which was terminated due to COVID-19-related disruptions.

TAK-062 TAK-062 is an engineered enzyme derived from kumamolisin, originally sourced from Alicyclobacillus sendaiensis and modified to enhance its ability to break down proteins. It specifically targets proline-glutamine (P-Q) dipeptide bonds and is designed to remain effective across the gastrointestinal tract's varying pH levels and in the presence of digestive protease. TAK-062 function is independent of the type of meal consumed. In-vitro studies have shown that a 900 mg dose of TAK-062 was well tolerated and capable of breaking down more than 99% of gluten (from 3 and 9 g doses) within 10 min [20]. The Illuminate-062 Study (NCT05353985) is a Phase 2 clinical trial aiming to evaluate the positive effect of TAK-062 on celiac-related symptoms and small intestinal damage due to gluten exposure. In particular, participants were invited to take TAK-062 in pill form thrice daily before a meal and fill out a daily symptom diary. Participants will eat a snack bar three times a week, sometimes containing a small amount of gluten. The study was suspended after an interim analysis and results are not available yet [21].

### Gluten sequestration

BL-7010 is a nonabsorbable polymer with a high molecular weight, that shows a strong affinity for gliadin [22] and blocks the absorption of immunogenic and toxic gluten peptides, potentially modulating the inflammatory and immune responses described in CeD. Laboratory studies showed that BL-7010 effectively reduces gliadin- or gluten-induced damage in cultured cells [23]. BL-7010 role was evaluated in the randomized, double-blind trial NCT01990885, which studied the safety of single and repeated doses in patients with controlled CeD. Although the trial stopped several years ago, its results have not been published yet.

Avian Immunoglobulin Y (AGY) are antibodies derived from the yolks of eggs laid by hyperimmunized hens. These antibodies are natural, cost-effective, and hygienic therapeutic agents with very low toxicity, except for individuals with egg allergies. When encapsulated, these IgY antibodies are referred to as AGY. Their primary therapeutic action involves binding to gliadin peptides found in food. A clinical trial (NCT01765647) involving 10 patients investigated AGY's potential to relieve symptoms of CeD (especially fatigue, headaches, and bloating). However, the results were inconclusive [24]. Another larger trial (NCT03707730), which has completed enrollment, is ongoing. This randomized, double-blind, placebo-controlled, crossover study includes 169 celiac disease patients on GFDs and aims to assess the safety and efficacy of AGY. The results are not yet available [25].

### Intestinal barrier repairing/tight junction modulators

Elevated zonulin levels are detectable in the earliest stages of CeD, suggesting their potential as early biomarkers for the condition [26^▪^]. Beyond zonulin-dependent mechanisms, studies have also identified innate changes in intestinal permeability in CeD patients that may play a role in the onset and progression of the disease [27].

Research has primarily focused on larazotide acetate (AT-1001), a synthetic octapeptide derived from the occludin zone toxin of Vibrio cholerae [28]. Larazotide acetate is a zonulin receptor inhibitor, reducing tight junction permeability in the intestinal epithelium. AT-1001 prevents tight junction opening and limits the passage of gluten peptides and the associated immune activation. Clinical trials have demonstrated that larazotide acetate is biologically well tolerated, and patients treated with it in combination with a GFD reported better symptom control than GFD alone. However, the change in intestinal permeability, measured through the urinary lactulose-to-mannitol ratio, did not significantly differ between the larazotide and groups [29]. In the NCT00492960 trial, 184 patients on a GFD received doses of 1, 4, or 8 mg of larazotide acetate three times daily, or placebo, along with 2.7 g of gluten for 6 weeks [30]. While the lactulose-to-mannitol ratio did not change significantly, the 1 mg dose notably reduced gluten-induced symptoms and the rise in antitissue transglutaminase antibodies. In another dose-ranging, placebo-controlled trial (NCT00362856), larazotide acetate reduced gluten-induced gastrointestinal symptoms at lower doses but was less effective at higher doses. The study highlighted variability in its impact on intestinal permeability while affirming its potential to lessen symptom severity [31].NCT03569007 [32] is a phase 3, randomized, double-blind, placebo-controlled, multicenter, outpatient study named CeDLara (Celiac Disease Larazotide). It evaluates the efficacy and safety of larazotide acetate for the relief of persistent symptoms in 525 adult patients with CeD on GFD. Although the study was completed in 2022, no results have been published yet. Therefore, larazotide acetate shows potential as a therapeutic agent for symptom relief in celiac disease patients on a GFD; however, further Phase 3 trials are needed.

### Transglutaminase inhibition

ZED1227 is a selective small-molecule inhibitor of tissue transglutaminase 2 (tTG2). The compound binds specifically to the active form of tTG2, creating a stable covalent bond with the cysteine residue in its catalytic core [33]. Clinical trials have demonstrated that ZED1227 is highly biologically safe, remaining effective and well tolerated at doses up to 500 mg. Schuppan *et al*. [34] reported findings from a phase 2, double-masked, placebo-controlled study investigating ZED1227. Participants received the drug daily over a 6-week period while undergoing a controlled gluten challenge of 3 g per day. The study's primary goal was to evaluate the reduction of gluten-induced mucosal damage, assessed through the villus height-to-crypt depth ratio. Preventing such mucosal damage is a key factor in maintaining long-term health in individuals with celiac disease, and this trial successfully achieved its primary objective. ZED1227 demonstrated a significant effect compared to placebo across all three tested doses (10, 50, and 100 mg). This outcome is significant considering that the study included a moderate gluten challenge (3 g daily versus the 12 g typical of a regular diet) over a relatively short timeframe [34]. ZED1227 is also known as TAK-227. In October 2022, Takeda Pharmaceutical Company entered into a collaboration and licensing agreement with Zedira and Dr Falk Pharma to advance the development and commercialization of TAK-227. Under this agreement, Takeda secured exclusive rights to develop and market the therapy in the United States and other specified territories, while Zedira and Dr Falk Pharma retained rights in Europe, Canada, Australia, and China. A Phase IIb trial, sponsored by Dr Falk Pharma, evaluated the efficacy and safety of ZED1227 compared to a placebo in individuals with CeD who continued to experience symptoms despite adhering to a GFD. This trial concluded at the end of September 2024 [35], and its results are anticipated to be released in the coming months.

### Gluten tolerization

In CeD, gluten enters the lamina propria of the intestinal mucosa, where tTG2 deamidates it. The modified gluten is then presented to CD4^+^ T cells by antigen-presenting cells (APCs), triggering immune responses that lead to intestinal mucosal damage. Targeting this antigen presentation process and modulating T-cell activation to induce immune tolerance presents a promising therapeutic approach. These strategies aim to reduce the activation of CD4^+^ T cells and the pathological response to gluten, fostering tolerance without necessarily breaking down or neutralizing gluten itself.

KAN-101 is a synthetic liver-targeting glycopolymer conjugated to a deaminated peptide derived from wheat alpha-gliadin. It promotes T-reg cell activation and inhibits CD4+ T cell immune responses. It is designed to induce immune tolerance to gliadin, specifically targeting individuals with the HLA-DQ2.5 genotype [36]. Administered intravenously, KAN-101 aims to modulate the immune system by selecting antigen-specific T cells, inducing anergy (nonresponsiveness) in these cells, and promoting regulatory T cell (Treg) activity. A Phase 1 study (NCT04248855) evaluated the safety and tolerability of KAN-101 in CeD patients adhering to GFD. Increasing doses (0.15–1.5 mg/kg) showed an acceptable safety profile with no dose-limiting toxicities or maximum tolerated dose identified. Rapid clearance and lack of accumulation with repeated dosing suggest potential for long-term use. Other ongoing Studies are NCT06001177 [37]: a Phase 2a multicenter, double-blind, placebo-controlled trial, evaluating whether KAN-101 can prevent gluten-induced histological changes in the duodenum of adults with CeD on a GFD, and NCT05574010 [38], a three-part, multicenter Phase 1b/2 study of KAN-101 in participants with CeD on GFD assessing the pharmacodynamic, safety, tolerability, and pharmacokinetics of KAN-101.

TAK-101 consists of gliadin encapsulated within negatively charged poly(DL-lactide-co-glycolic acid) nanoparticles. These nanoparticles are administered intravenously and are taken up by APCs in the liver and spleen. Once internalized, the nanoparticles present gliadin to gliadin-specific T cells, promoting immune tolerance through mechanisms such as T cell anergy and the activation of regulatory T cells. NCT03486990 was the initial trial assessing the safety and tolerability of TAK-101. Results demonstrated that the therapy was well tolerated, with no serious adverse events or significant changes in vital signs and laboratory parameters, confirming its acceptable safety profile. NCT03738475 is a double-blind, randomized, placebo-controlled trial focused on the drug's efficacy in mitigating gluten-induced immune activation in CeD patients adhering to a GFD. During a 14-day gluten challenge, 33 participants received TAK-101. The primary endpoint measured changes in gliadin-specific interferon gamma (IFN-γ)-producing cells, which are a key marker of CeD's immune response. TAK-101 administration led to an 88% reduction in IFN-γ-producing cells compared to placebo, a statistically significant outcome (*P* = 0.006), indicating a potent immunomodulatory effect. Among the ongoing studies, NCT04530123 aims to further evaluate TAK-101's efficacy in reducing gluten-related symptoms and immune activation during a gluten challenge.

Nexvax2 consists of three synthetic peptides containing six HLA-DQ2.5-restricted immune-dominant T cell epitopes. It functions similarly to bystander inhibition, promoting immune tolerance [39]. However, the subsequent clinical trial was discontinued because improvements in symptoms and serological markers did not meet the desired criteria. An initial Phase 1 clinical trial [40] (NCT00879749) evaluated Nexvax2, a gluten peptide-based, antigen-specific immunotherapy designed to desensitize T cells and render them nonreactive to gluten and confirmed its bioactivity- However, additionally, a Phase 2 trial (NCT03644069) assessing its impact on patient-reported outcomes was prematurely halted after an interim analysis revealed that Nexvax2 was ineffective in alleviating acute gluten-induced symptoms [41].

### Lymphocyte migration inhibitors

CCX282-B is an orally administered antagonist of C-C chemokine receptor type 9 (CCR9) found on circulating lymphocytes and plays a critical role in directing these immune cells to the intestines. C-C chemokine ligand 25 (CCL25) is produced by intestinal epithelial cells and is upregulated during inflammation [42]. A Phase 2 clinical trial (NCT00540657), conducted in a double-blind, randomized, placebo-controlled design, explored the efficacy of CCX282-B in mitigating the effects of gluten exposure in patients with CeD. Ninety participants were enrolled, with half receiving 250 mg of CCX282-B twice daily for 13 weeks. The primary endpoint was to assess the impact of CCX282-B versus placebo on the villus height to crypt depth ratio in small intestinal biopsy samples taken before and after gluten exposure. Secondary outcomes included the evaluation of mucosal inflammation, celiac-related antibody levels, and symptom severity. The trial concluded in 2008; however, the findings were not published.

Teriflunomide works by inhibiting de-novo pyrimidine synthesis, thereby exerting a cytostatic effect that limits lymphocyte proliferation [43]. A Phase 2a double-blind, randomized, placebo-controlled trial (NCT04806737) assessed the efficacy and tolerability of a 14-day course of teriflunomide in 15 individuals with CeD who underwent a 3-day gluten challenge. However, as with similar studies, the results of this trial have not yet been published.

### Anti-IL-15 treatment

The pro-inflammatory cytokine IL-15, recognized as a pivotal factor in the development of CeD, is secreted by APCs and intestinal epithelial cells. It plays a central role in activating and promoting the proliferation of IELs, primarily CD8^+^ T cells, which attack the intestinal epithelium and contribute to villus atrophy [44].

AMG714, the first mAb designed to target IL-15, has been studied as a potential therapy for CeD. In one clinical trial, participants on a long-term GFD received six subcutaneous doses of AMG714 (150 or 300 mg) over 10 weeks, administered every two weeks. From weeks 2 to 12, patients were exposed to daily gluten intake (2–4 g) [18]. While the 150 and 300 mg doses did not prevent gluten-induced intestinal damage compared to the placebo group, the 300 mg dose was associated with a smaller increase in IEL levels and less severe symptoms. Another study [45] focused on AMG714's effects in individuals with refractory type 2 CeD. Over a 10-week period, the treatment showed no significant improvement in the primary outcome of reducing abnormal IEL counts when compared to placebo. However, 5-year follow-up data from a single center on 11 patients with type 2 refractory CeD coeliac disease, shows clinical remission in all patients treated with AMG 714, mucosal recovery, and improvement in clonality in most cases [46]. The ongoing *NCT03439475* trial [47] evaluates AMG 714 in adult patients with biopsy-proven refractory type 2 CeD who have failed all available treatment options and do not have enteropathy-associated T-cell lymphoma (EATL).

PRV-015 is a mAb against IL-15. An ongoing Phase 2b trial (NCT04424927) [48], is evaluating the efficacy and safety of three-dose regimens of PRV-015 in patients with non-responsive CeD as an adjunct to a GFD.

### Probiotics

In-vitro studies have demonstrated the ability of VSL#3 probiotic formulation to break down gliadin polypeptides [49]. However, a multicenter Italian study on 45 CeD patients showed no significant changes in symptom severity in fecal microbiota composition using VSL#3 [50]. Another study investigated the effects of *Bifidobacterium natren life* start (NLS) [51] in 22 untreated CeD patients, revealing that the probiotics alleviated gastrointestinal symptoms and produced some immunologic changes such as the increase in serum macrophage inflammatory protein-1β but did not modify abnormal intestinal permeability. A more recent randomized, cross-over, double-blind, placebo-controlled trial evaluated the effect of *Bifidobacterium infantis NLS super strain* (NLS-SS) on persistent gastrointestinal symptoms in patients with CeD on GFD for at least 2 years [52]. The study demonstrated the improvement in specific gastrointestinal symptoms in CeD patients treated with *B. infantis* NLS-SS and a shift in stool microbiota profile with decreased abundance of *Ruminococcus sp*. and *Bifidobacterium adolescentis.*

## DISCUSSION

The management of CeD currently relies on a GFD, which is generally well tolerated. While the GFD remains effective, it poses social, psychological, and practical challenges. Future treatments should improve efficacy while maintaining safety comparable to a GFD. New therapeutic approaches targeting the underlying mechanisms of CeD have emerged in order to ease the burden of dietary restrictions and improve patients’ quality of life. These include strategies to modify gluten processing in the gut, block gluten-triggered immune responses, or restore immune tolerance to gluten. This field of research is active, with ongoing and completed trials aiming to validate these methods. ZED1227 (also known as TAK-227), is a therapeutic agent that has garnered significant attention. If successful in the ongoing clinical trials, TAK-227 could represent a valid pharmacological treatment for celiac disease, offering an alternative to the strict GFD. Although phase II trials have shown promise for several therapeutic targets, broader validation through phase III trials is needed. Clinical trials often show discrepancies between symptoms, serological findings, and mucosal biopsy results, emphasizing the need for further research. In many cases, final results and pitfalls had not been published yet. Moreover, the studies conducted so far exhibit several limitations in terms of both study design and methodology. In most cases, the protocols enrolled CeD patients with gastrointestinal symptoms and evidence of duodenal mucosal damage, which often suggests poor adherence to a GFD, likely persisting also during the trial. It would be more beneficial perhaps to enroll patients with strict adherence to a GFD, negative antibody levels, and no mucosal damage, assigning them to either the drug-treated or placebo arm. Alternatively, patients with a recent diagnosis of CeD (e.g., within the past six months), who are following a GFD but have not yet shown clear normalization of duodenal mucosa, could be included. Data currently available regarding the use of these new therapies in patients with refractory CeD are also still limited, and only a few registered trials are currently underway.

Long-term safety, individual variability in response, and cost-effectiveness must also be addressed. In fact, new treatments are likely to be costly, potentially adding to the financial burden already posed by a GFD.

## CONCLUSION

Integrating new therapies into clinical practice will require collaboration among healthcare professionals and patient education. Balancing patient autonomy in choosing between dietary and medical treatments with the financial feasibility of widespread adoption is essential. Future research should focus on personalized treatment plans, the long-term safety of emerging drugs, and adjunctive therapies to enhance life quality.

## Acknowledgements

*None.*

### Financial support and sponsorship

*None.*

### Conflicts of interest

*The authors declare no conflict of interest.*

## REFERENCES AND RECOMMENDED READING

Papers of particular interest, published within the annual period of review, have been highlighted as:

▪ of special interest
▪▪ of outstanding interest
